# Gene expression network analyses in response to air pollution exposures in the trucking industry

**DOI:** 10.1186/s12940-016-0187-z

**Published:** 2016-11-03

**Authors:** Jen-hwa Chu, Jaime E. Hart, Divya Chhabra, Eric Garshick, Benjamin A. Raby, Francine Laden

**Affiliations:** 1 Section of Pulmonary, Critical Care and Sleep Medicine, Department of Internal Medicine, Yale University School of Medicine, New Haven, CT USA; 2 Channing Division of Network Medicine, Brigham and Women’s Hospital, Harvard Medical School, Boston, MA USA; 3 Department of Environmental Health, Harvard T.H. Chan School of Public Health, Boston, MA USA; 4 Pulmonary, Allergy, Sleep, and Critical Care Medicine Section, VA Boston Healthcare System, Boston, MA USA; 5 Division of Pulmonary and Critical Care Medicine, Brigham and Women’s Hospital, Harvard Medical School, Boston, MA USA; 6 Department of Epidemiology, Harvard T. H. Chan School of Public Health, Boston, MA USA

**Keywords:** Air pollution, Trucking industry, Gene expression, Network analysis

## Abstract

**Background:**

Exposure to air pollution, including traffic-related pollutants, has been associated with a variety of adverse health outcomes, including increased cardiopulmonary morbidity and mortality, and increased lung cancer risk.

**Methods:**

To better understand the cellular responses induced by air pollution exposures, we performed genome-wide gene expression microarray analysis using whole blood RNA sampled at three time-points across the work weeks of 63 non-smoking employees at 10 trucking terminals in the northeastern US. We defined genes and gene networks that were differentially activated in response to PM_2.5_ (particulate matter ≤ 2.5 microns in diameter) and elemental carbon (EC) and organic carbon (OC).

**Results:**

Multiple transcripts were strongly associated (p_adj_ < 0.001) with pollutant levels (48, 260, and 49 transcripts for EC, OC, and PM_2.5_, respectively), including 63 that were statistically significantly correlated with at least two out of the three exposures. These genes included many that have been implicated in ischemic heart disease, chronic obstructive pulmonary disease (COPD), lung cancer, and other pollution-related illnesses. Through the combination of Gene Set Enrichment Analysis and network analysis (using GeneMANIA), we identified a core set of 25 interrelated genes that were common to all three exposure measures and were differentially expressed in two previous studies assessing gene expression attributable to air pollution. Many of these are members of fundamental cancer-related pathways, including those related to DNA and metal binding, and regulation of apoptosis and also but include genes implicated in chronic heart and lung diseases.

**Conclusions:**

These data provide a molecular link between the associations of air pollution exposures with health effects.

**Electronic supplementary material:**

The online version of this article (doi:10.1186/s12940-016-0187-z) contains supplementary material, which is available to authorized users.

## Background

Air pollution exposures, have been associated with a number of adverse health effects, including greater morbidity and mortality risks for cardiopulmonary diseases, and increased risk of lung cancer [1–6]. However, the underlying biological mechanisms have not been fully elucidated. Human studies of global changes in gene expression following controlled exposures [7], or using in vitro models [8, 9] have provided some insights in this regard, yet few studies have rigorously assessed the impact of air pollution on gene expression in real-life settings. For example, though observational studies have been conducted in individuals from geographic regions with differing levels of air pollution have suggested associations, [10] studies with more refined exposure measures have not been performed.

In this study, we characterized the cellular response induced by traffic-related air pollution exposures in a population of non-smoking US trucking industry employees. We performed genome-wide gene expression microarray analysis using whole blood RNA sampled at three time-points during the work week. We integrate these data with micro-environmental measures of occupational exposure to three pollutants –particulate matter ≤ 2.5 microns in aerodynamic diameter (PM_2.5_), elemental carbon (EC), and organic carbon (OC) in PM_1.0_ (particulate matter with a diameter of ≤1.0 μm). Our objective was to identify the genes and gene networks differentially activated in response to these exposures.

## Methods

### Study population

A total of 95 subjects were recruited from 10 trucking terminals in the northeastern US (CT, MA, MD, NJ, NY, and PA). The participants were workers whose job duties were characterized by different patterns of exposure: pick-up and delivery (P&D) drivers, with regular exposures to traffic; loading dock workers with regular exposures to propane forklifts and episodic exposures to diesel trucks and other vehicles in the terminal yard; office workers with no occupational traffic related exposures, and combination workers, who performed the job duties of a P&D driver or a dock worker, as needed.

The measurements took place between February 2009 and October 2010. Each subject was enrolled on the first day of the workweek following at least two days off. Whole blood samples were collected using PaxGene RNA tubes, three times from each subject: (1) before the first shift of the workweek (first day, AM draw); (2) at the end of the first shift (8–12 h later) on the same day (first day, PM draw); and (3) at the end of the last work shift of the same workweek (last day, PM draw). This design allowed us to assess the cross-shift effects after returning from work after at least 2 days off, and the cross-week effects (i.e., over 2–5 days). Our primary analyses were restricted to the 63 Caucasian non-smoking male workers with at least a single blood sample available. The majority of participants were excluded for being current active smokers (*n* = 21) given the known effects of smoking on gene expression [11] four participants were unable to provide a sufficient blood sample, two reported an active illness (cold or flu) at the time of blood draw, and one female and 7 non-white men were excluded. The final data set includes a total of 165 samples.

### Measurement of traffic exposures

Micro-environmental samples of PM_2.5_, and EC and OC in PM_1.0_ were collected over the full workweek (24 h/day for 6–9 days) at each of the 10 terminals. Twelve-hour area samples were collected indoors in office spaces and terminal docks. Samples also were collected in the truck cabs of participating drivers during their work shifts on their first and last day of work. Detailed information on the exposure assessment for each of the three pollutants is described elsewhere [12]. Briefly, EC and OC were measured by collecting PM_1.0_ on a 22-mm quartz tissue filter, preceded by a precision machined cyclone separator (SCC1.062 Triplex, BGI, Inc., Waltham, MA), which was then analyzed with thermal-optical carbon analyzer using the NIOSH 5040 method [13]. PM_2.5_ was collected on a pre-weighed 37-mm Teflon filter (with a pore diameter of 0.2 μm) after passing through a precision-machined cyclone pre-selector to remove particles greater than 2.5 μm in aerodynamic diameter. After collection, the filter was reweighed to obtain the mass of PM_2.5_ collected. The method was consistent with the EPA PQ200 Federal Reference Method [14, 15]. For each participant on each day, exposures to PM_2.5_, EC, and OC were assigned as a weighted average of the time spent in each work location.

### Gene expression data

Blood samples were stored at 4 °C on the day of collection until they were shipped overnight each day to our blood processing laboratory in Boston, MA in an insulated container with a cooler pack to keep samples chilled. Upon arrival, RNA was extracted using the Qiagen RNAEasy extraction kit, according to protocol and then stored at −80 °C until analysis. Gene expression profiling was conducted using the Illumina HumanHT-12 v4 Expression BeadChip, with RNA labeling and array hybridization performed according to protocol. Image capture was performed using the Illumina BeadArray Reader. Standard QC and preprocessing procedures were applied to remove failed samples (*n* = 2). Standard background correction and normalization procedures (Variance-Stabilizing Transform, [16]) were applied using the R package *lumi*. The final data set included information from 47,295 probes on 165 samples from 63 subjects.

### Statistical analysis

To maximize the power of our repeated measures of gene expression, we employed a mixed effect model that considered gene expression measures from all three blood draws, with the form:

$$ \mathrm{Expression}=\mathrm{Exposure}+\left(1\left|\mathrm{subject}\right.\right)+\mathrm{Confounders}; $$

where the expression measurements were treated as repeated measures. Each exposure was considered separately, and to estimate the impact of long-term exposures, we used the average of the exposure measures from the first and last work shift for each participant. Personal factors were considered as potential confounders including age, and body mass index (BMI). Job title and terminal were not considered as confounders as they were assumed to be proxies for our measured exposures. BMI was not associated with either the exposure or the outcome, therefore the only covariate included in the final model was age. Statistical significance was determined by estimating the False Discovery Rate (FDR) by permutation testing, to correct for possible *p*-value inflation introduced by the covariance of repeated measures. We also tested for the cross-shift effects (blood draw 1 vs. blood draw 2) and the cross-week effects (blood draw 1 vs. blood draw 3) within each subject, using the difference in the expression measurement between the blood draws as the response variable.

Gene expression changes to environmental perturbations are thought to arise through coordinated responses of specific gene networks that are often difficult to appreciate through single gene testing. We therefore applied Gene Set Enrichment Analysis (GSEA) using GSEA software from the Broad Institute to identify subsets of genes with shared function that were altered by exposure to vehicle exhaust from the Molecular Signature database (MSigDb [17]), which is a collection of annotated gene sets for GSEA analysis. The gene sets are categorized into different collections (C1-C7), and here we considered six functional categories of gene sets: C2 (curated gene sets), C3 (motif gene sets), C4 (computational gene sets), C5 (GO gene sets), C6 (oncogenic signatures), and C7 (immunologic signatures). The gene set enrichment analyses are based on a list of genes ranked by effect sizes from the linear mixed effect models for all three types of exposures were performed. Those gene sets that were significantly enriched for all three types of exposures (EC, OC, PM_2.5_) were marked for further functional annotation. Significance was claimed at an FDR of 25 %, as recommended for GSEA. For these enriched gene sets, we also identified the genes that contributed most to the enrichment (“leading edges”). In addition, we also performed connectivity map analysis to identify additional genes connected to the genes correlated with exposure levels. Finally, we performed GSEA analyses on data from two other air pollution related Gene Expression Omnibus (GEO) datasets (GSE7462 [7] and GSE7543 [10]) to assess the generalizability of our results.

GSE7462 [7] is from a crossover, double-blind study of the effects of diesel exhaust inhalation compared to fresh air exposure on peripheral blood mononuclear cells (*n* = 23); and GSE7543 [10] is a study of differences in expression in peripheral blood samples collected from two regions of the Czech Republic with markedly different levels of pollution (*n* = 71).

## Results

Selected characteristics of the study subjects, and the mean exposure levels of PM_2.5_, EC and OC observed over the 5-day workweek, are presented in Table 1. The participants were 50.5 years old (SD = 8.4) on average, 52.4 % were former smokers, and pick-up and delivery drivers were the largest job group sampled (46.0 %). Out of the three pollutants, EC and PM_2.5_ were modestly correlated (*r* ^2^ = 0.38), while EC-OC and PM_2.5_-OC were not (*r* ^2^ = 0.02 and 0.07, respectively). We found no systematic difference in expression measurements or other demographic variables between the subjects with complete blood samples at all three collection times and the subjects with missing data (data not shown).

**Table 1:** Characteristics of the 63 white male trucking industry workers

	Total
Total no.	63
Age (years, mean ± SD)	50.5 ± 8.4
Past smoker (no. (%))	33 (52.4)
Primary job title (no.(%))	
Pick-up and Delivery Driver	29 (46.0)
Dockworker	12 (19.1)
Officeworker	15 (23.8)
Combination Workers^a^	7 (11.1)
Workweek average exposure (μg/m^3^, mean ± SD)	
PM_2.5_	11.5 ± 5.1
EC	0.6 ± 0.4
OC	8.8 ± 3.0

^a^Combination workers perform the jobs of pick-up and delivery drivers or dockworkers as needed

**Fig. 1:**
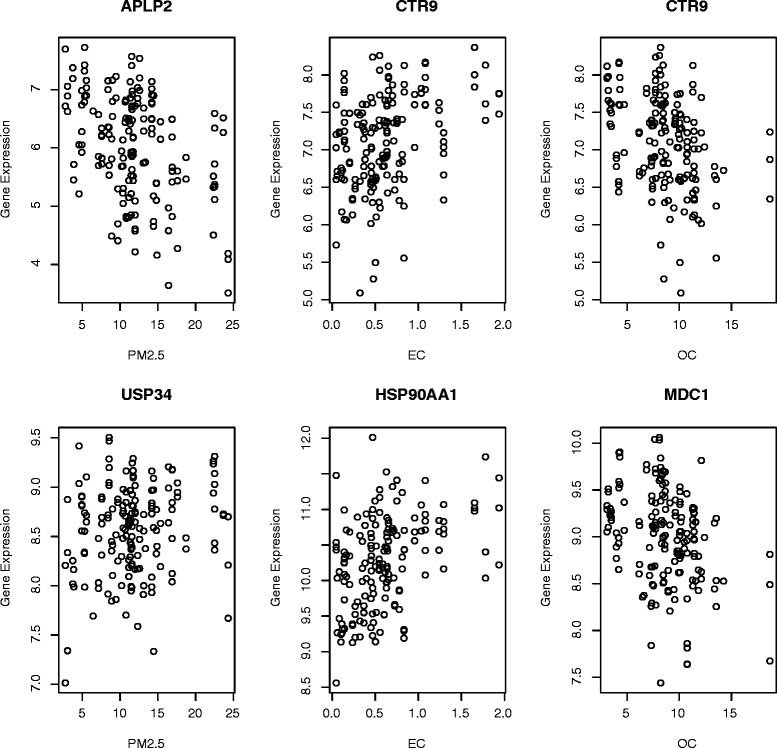
Dot plots of most important expression profiles from core gene set. Left: Dot plots for genes most correlated with PM_2.5_ (APLP2 and USP34); Middle: Dot plots for genes most correlated with EC (CTR9 and HSP90AA1); Right: Dot plots for genes most correlated with OC (CTR9 and MDC1)

For the gene-level differential expression analysis, the tests for cross-shift and cross-week effects did not yield any significant results. Therefore we focus on the results from linear mixed-effect model. QQ plots contrasting the observed with permuted *p*-value distributions for the linear mixed effect model analyses are presented in Additional file 1: Figure S1, demonstrating excessive deviations of the observed results from expectation. We estimated the genomic inflation factor lambda for each analysis (EC = 1.09, OC = 1.39, and PM_2.5_ = 1.15), to enable adjustment of our results for unobserved technical biases. Multiple transcripts were strongly correlated (padj < 0.001) with week-long average pollutant levels (EC *n* = 48, OC *n* = 260, PM_2.5_ *n* = 49, see Fig. 1 for examples of most strongly correlated genes for each exposure), including 67 that were strongly correlated with at least two of three exposure measurements (See Additional file 2: Table S1), though no individual genes met our *a priori* threshold of statistical significance at an FDR < 0.1.

**Fig. 2:**
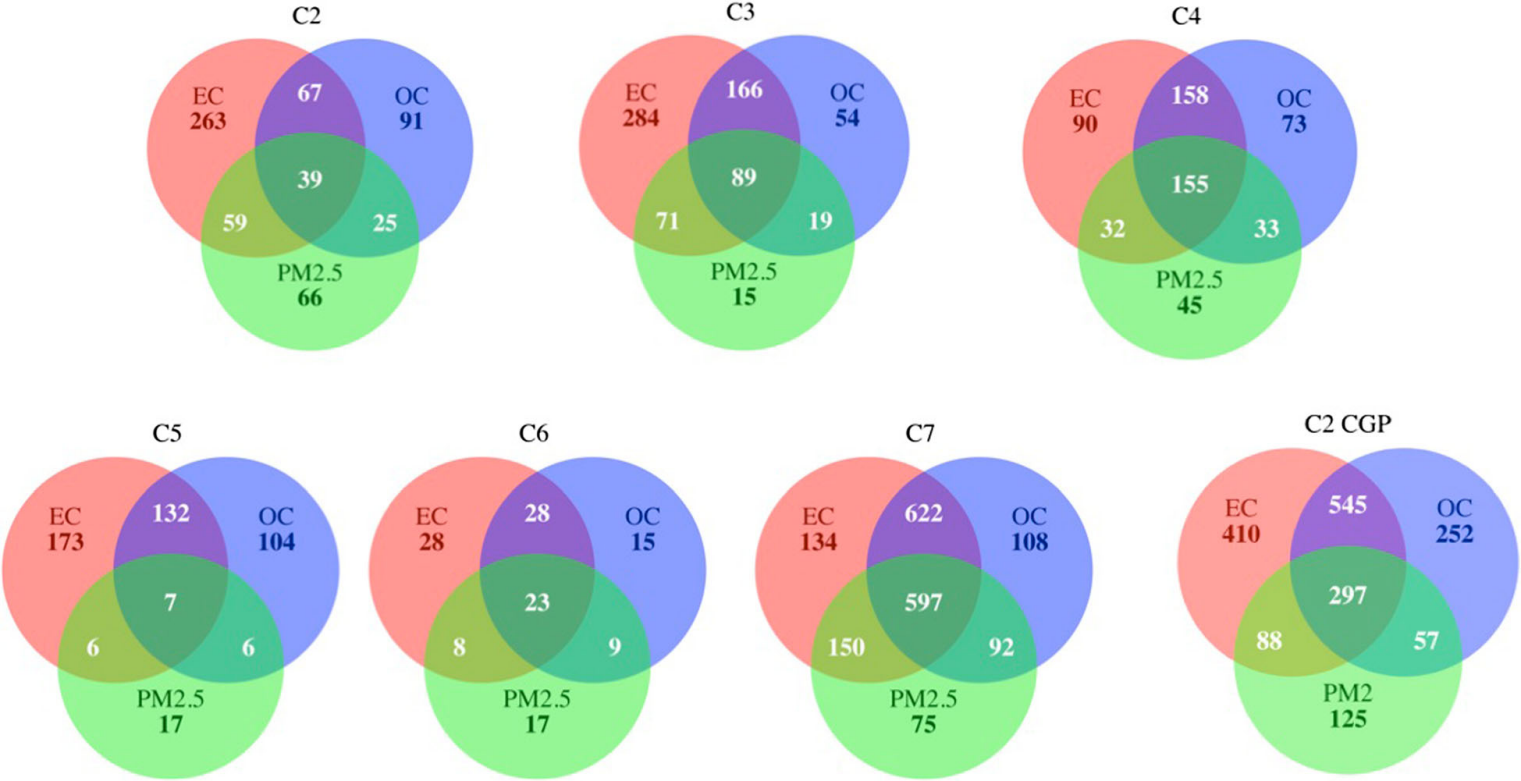
Venn Diagrams for gene sets. The numbers of the gene sets that were significantly enriched with FDR < 0.25 and nominal *p*-values < 0.05. Note: C2 = curated gene sets (excluding CGP gene sets, see below), C3 = motif gene sets, C4 = computational gene sets, C5 = GO gene sets, C6 = oncogenic signatures, C7 = immunologic signatures, CGP = Chemical and genetic perturbations

In contrast to the gene level analysis, GSEA revealed widespread differential responses with long-term exposures. A total of 6019 gene sets were significantly enriched (FDR < 0.25 and nominal *p*-value < 0.05) for any of the three exposure types (See Fig. 2), including 2384 gene sets from the C2 collection, 698 sets from the C3 collection, 586 from the C4 collection, 445 from the C5 collection, 128 from the C6 collection, and 1778 from the C7 collection. EC exposure was consistently correlated with the greatest number of enriched gene sets across all collections. Most notable was the great degree of gene set enrichment overlap across exposure types. Overall, 59.5 % (3580 of 6019) of gene sets were implicated in more than one exposure response, including 82.2 % of C7 collection sets, 64.5 % of C4 sets, 53.1 % of C6 sets, 49.4 % of C2 sets, 49.4 % of C3 sets, and 33.9 % of C5 sets (Fig. 2). Further inspection revealed that 20 % (1207) of gene sets were enriched across all three pollutants (*p* < 10^−16^ for any sharing between pollutants, *p* < 10^−16^ for sharing across all three pollutants).

From the regression and GSEA analyses, we defined a core set of 262 genes whose expression was modified by occupational exposure to vehicle exhaust and that were most frequently represented in enriched gene sets that emerged from the GSEA (Additional file 2: Table S1). This core gene set included the 20 genes most differentially expressed for each pollution measure (OC, EC, and PM), those that were differentially expressed by two or more measures, genes that were enriched in at least 10 gene sets per MSigDb collection, and genes that were enriched in at least 20 sets common to all three exposure measures. To assess whether members of this core set were specific to our study, or have been implicated by others, we formally tested whether this core gene set was overrepresented in two previously published, independent air pollution gene expression datasets available through GEO.. We observed statistically significant enrichment of our selected gene set in both datasets (*p* < 10^−16^ for each). Of the 262 genes, 114 were within the leading edge for the GSE7462 dataset, and 64 for GSE7543, with 25 genes common to both (Table 2). This common set of leading edge genes included multiple genes implicated in the interrelated processes of DNA binding (LEF1, MLH1, RBM5, STAT1, CITED2, APLP2, DDX3Y, ZNF589), metal binding (MAN1A1, PRIM1, RBM5, STAT1, ZFP161, and ZNF589), apoptosis regulation (CITED2, MLH1, RBM5, STAT1), and cancer pathways (HSP90AA1, LEF1, MLH1, STAT1).

**Table 2:** List of 25 genes in the core gene set that were differentially expressed in two independent GEO data sets

Gene symbol	Gene name	Number of interactions	Number of pathways	Mean (SD) log2 expression			*p*-value			
				Trucker	Dock	Office	ANOVA	PM_2.5_	EC	OC
GNAQ	Guanine nucleotide binding protein, q polypeptide	15	18	5.96 (1.17)	7.04 (0.91)	6.15 (1.74)	0.0004	0.1535	0.0116	0.0017
CTR9	Ctr9, Paf1/RNA polymerase II complex component	13	7	7.01 (0.64)	7.44 (0.49)	7.10 (0.61)	0.0037	0.5095	6.11E-06	5.59E-06
HSP90AA1	Heat shock protein 90 kDa alpha, class A member 1	12	0	10.24 (0.66)	10.43 (0.35)	10.13 (0.74)	0.1687	0.8814	1.36E-08	0.0353
MLH1	mutL homolog 1	10	19	8.66 (0.29)	8.83 (0.21)	8.65 (0.39)	0.0184	0.0037	0.0025	0.0547
FNBP4	Formin binding protein 4	10	23	10.36 (0.49)	10.86 (0.29)	10.44 (0.62)	1.10E-05	0.1515	0.0063	0.0004
ACP1	Acid phosphatase 1	10	13	8.71 (0.52)	9.22 (0.28)	8.69 (0.56)	3.53E-06	0.0107	1.95E-06	0.0003
MDC1	Mediator of DNA-damage checkpoint 1	10	17	8.93 (0.49)	9.32 (0.35)	8.93 (0.54)	0.0004	0.1663	0.0530	5.75E-05
LEF1	Lymphoid enhancer-binding factor 1	9	42	11.17 (0.45)	11.41 (0.32)	11.00 (0.46)	0.0006	0.0291	0.0096	0.0001
LPIN1	Lipin 1	9	28	9.69 (0.52)	10.17 (0.36)	9.78 (0.65)	9.96E-05	0.1880	0.0006	0.0005
RBM5	RNA binding motif protein 5	9	15	11.73 (0.30)	12.02 (0.22)	11.77 (0.39)	4.36E-05	0.0333	0.0101	0.0008
MAN1A1	Mannosidase, alpha, class 1A, Member 1	8	4	5.26 (0.97)	5.85 (0.75)	5.46 (1.06)	0.0138	0.2408	2.35E-05	2.06E-06
ZFP161	Zinc finger protein 161	8	17	8.08 (0.51)	8.28 (0.26)	8.05 (0.60)	0.1081	0.1419	0.0046	0.0030
HSPA8	Heat shock 70 kDa protein 8	7	21	12.49 (0.24)	12.66 (0.17)	12.47 (0.32)	0.0031	0.0096	1.81E-05	0.0081
PRIM1	Primase 1	6	39	7.70 (0.40)	7.88 (0.24)	7.56 (0.44)	0.0045	0.0632	3.47E-05	0.1771
STAT1	Signal transducer and activator of transcription 1	6	0	11.86 (0.60)	11.49 (0.34)	11.79 (0.51)	0.0066	0.6718	0.0821	2.14E-06
CITED2	Cbp/p300-interacting transactivator 2	4	19	9.62 (0.48)	9.84 (0.38)	9.70 (0.42)	0.0728	0.0201	0.0069	0.0112
APLP2	Amyloid beta precursor-like protein 2	4	0	6.01 (0.98)	5.84 (0.65)	6.21 (0.77)	0.2322	5.65E-08	0.3388	0.8264
DDX3Y	DEAD box polypeptide 3, Y-linked	4	16	2.48 (1.72)	2.84 (1.48)	2.70 (1.77)	0.5375	0.0574	0.0386	0.0191
HLA-DMA	Major histocompatibility complex, class II, DM alpha	4	0	12.50 (0.46)	12.18 (0.44)	12.45 (0.54)	0.0069	0.7922	7.36E-06	9.93E-06
DOCK9	Dedicator of cytokinesis 9	2	18	2.95 (1.48)	4.37 (1.19)	3.25 (1.82)	7.74E-05	0.1116	0.0001	0.0002
EML3	Echinoderm microtubule associated protein like 3	2	17	11.70 (0.39)	11.56 (0.33)	11.57 (0.34)	0.0787	0.0175	0.0133	0.0006
GPBAR1	G protein-coupled bile acid receptor 1	2	0	10.24 (0.60)	9.91 (0.48)	10.31 (0.81)	0.0227	0.9312	0.0002	7.27E-07
USP34	Ubiquitin specific peptidase 34	2	22	8.49 (0.39)	8.89 (0.31)	8.55 (0.58)	5.32E-05	0.0366	0.0095	0.0077
ZNF589	Zinc finger protein 589	1	0	9.00 (0.49)	9.02 (0.36)	8.66 (0.40)	0.0004	3.60E-08	0.2132	0.5830
CTAGE5	CTAGE family, member 5 pseudogene	0	0	3.26 (1.05)	4.10 (0.99)	3.68 (1.27)	0.0008	0.5163	0.5885	0.0008

**Fig. 3:**
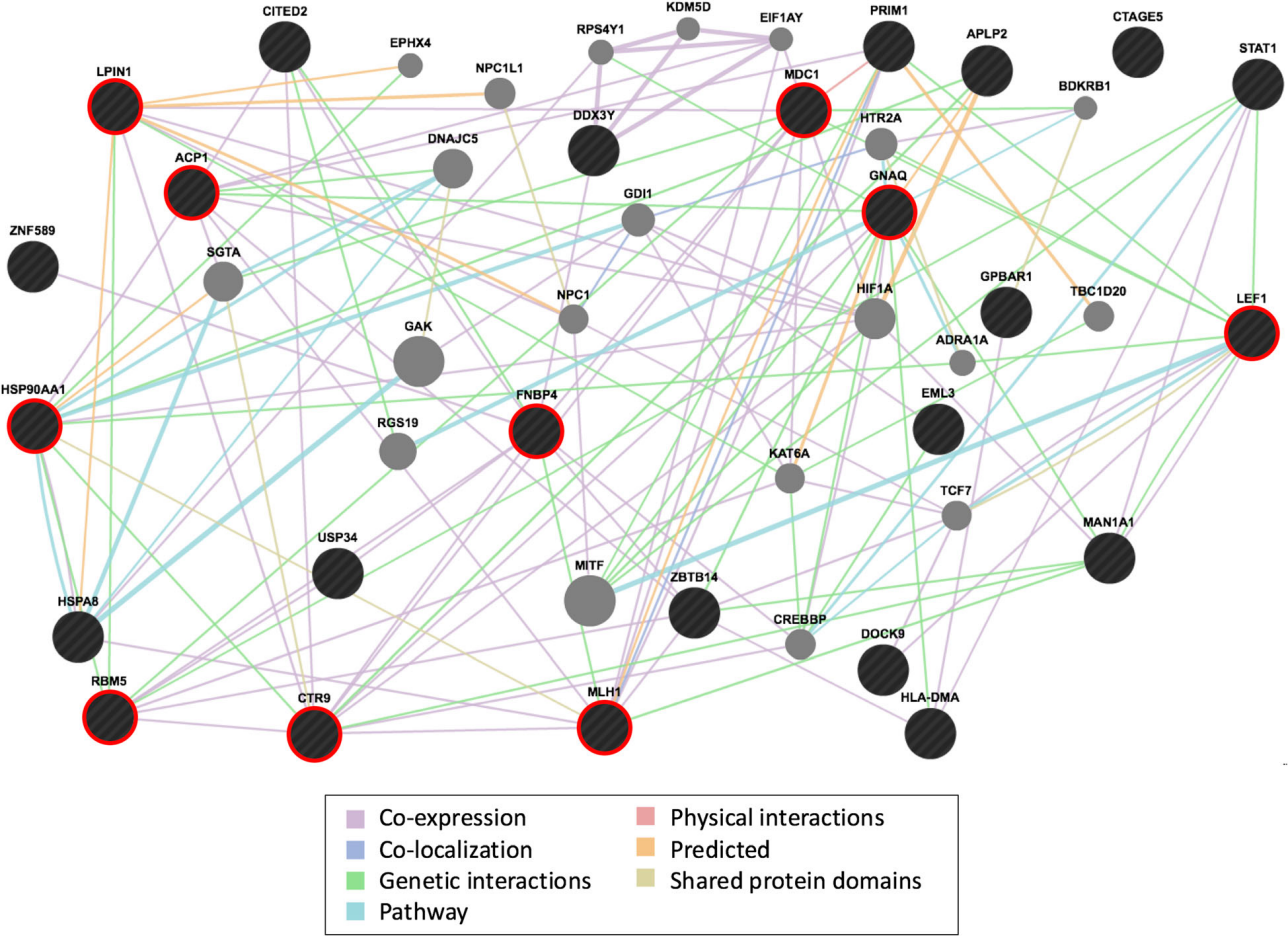
Interconnectivity of particulate induced genes

These 25 genes, which were derived from our expression analyses of three exposure measures and were also enriched in the two independent GEO datasets, represent the most highly reproducible (i.e. most robust) gene set to emerge from our analysis. To determine the interrelationship among this core set of genes, we applied GeneMANIA network analysis [18], the results of which are displayed in Fig. 3. The derived network consisted of 24 of the 25 core genes (the sole core gene not incorporated into the network, CTAGE5, is a known pseudogene) and 20 additional genes that were pulled in by GeneMANIA. Though these 44 genes have been implicated in numerous biological and cellular processes, several processes were statistically significantly overrepresented among these lists, including those of DNA binding (13 of 44 genes, *p* = 0.01), cell surface receptor linked signal transduction (11 genes, *p* = 0.01), and cancer (8, *p* = 1.1 × 10^−4^). The derived network demonstrated substantial evidence of interconnectivity: a total of 131 gene-gene interactions were observed, with each of the 25 core genes interacting on average with 6.95 other genes (range 1–15). Genes (nodes) with the highest number of interactions included GNAQ (15 connections), CTR9 (13), HSP90AA1 (12), MLH1, FNBP4, ACP1, MDC1 (each with 10 connections), and LEF1, LPIN1, and RBM5 (9 connections each). Together, these ten hub genes linked to all but two of the other genes in the network, and five (ACP1, HSP90AA1, LEF1, MLH1, and RBM5) are common to the major cancer-related pathways identified above (DNA, metal binding, and apoptosis regulation).

## Discussion

This study represents the first attempt to characterize the molecular impact of air pollution using micro-environmental measures of exposure. Four primary observations were made. First, GSEA demonstrated widespread evidence of pathway-specific changes in gene expression, with 6019 gene sets demonstrating enrichment for traffic-related air-pollution correlated genes. The enriched gene sets implicated a range of cellular responses and pathways. Several – including oxidative stress responses [19], interferon-mediated in amatory responses to viral infection [20], and hypoxemia-induced responses [21] – are known response mechanisms for dealing with the likely biological consequences of air pollutant exposures. Second, we found that despite the relatively low within-subject correlation of the measures of exposure, there was a striking degree of shared gene set enrichment across pollutants (59.5 % for any sharing, 20 % for sharing across all three exposures). The degree of overlap across the exposures, while far more than would be expected by chance, was not surprising because the three exposure metrics (EC, OC and PM_2.5_) are representing different properties of the same source. These overlapping gene sets implicated a core set of genes and pathways, suggesting a set of common molecular responses to such exposure. In addition, inspection of the gene memberships within the leading edges of these overlapping gene sets suggested evidence of overlap of critical genes, both across pathways and across pollutants. However, the 80 % of non-overlapping gene sets also suggest independent mechanisms may be more related to individual exposures. Third, our connectivity analysis defined a sub-network of interconnected genes at the heart of this shared response. Though several individual components of the network have been previously implicated in anti-oxidative and other protective cellular responses to pollution [22], our findings focused greater attention on these genes and their neighbors as central role players underlying these responses. Finally, we demonstrated the generalizability of our results beyond the trucking industry to the general population by demonstrating that the core set of overlapping genes that emerge from our analyses were also overrepresented in two independent air pollution expression datasets.

The major finding of our analysis is the characterization of a core network of interconnected genes common to all three air pollution measures (Fig. 3) that also form a common gene set that is enriched in two previously published air pollution-expression datasets. This core network consists of genes that are members of fundamental cancer-related pathways, including those related to DNA and metal binding, apoptosis regulation, and cell surface receptor linked signal transduction. Of potential greatest significance was the observation that 10 of the core genes formed connections with all but 2 of the 44 genes making up the network. Among these 10 hubs are 5 – ACP1, HSP90AA1, LEF1, MLH1, and RBM5 - common to the major identified cancer-related pathways. Though these genes have promiscuous function, they are all implicated in tumor pathobiology, providing a potential etiological link between the known associations of chronic air pollution exposure and lung cancer mortality [2, 4], including studies assessing diesel exhaust exposure in the trucking industry and other populations [6, 23, 24]. For example, acid phosphatase 1 (ACP1), whose expression was strongly correlated with PM_2.5_ exposure in our cohort and was a leading edge member of 13 gene sets enriched across all three exposure types, is a low-molecular-weight protein tyrosine phosphatase (LMW-PTP) with both pro- and anti-oncogenic functions (reviewed in [25]). Two common isoforms have been described, both were correlated with PM_2.5_ exposure in our dataset (*p* = 4.7 × 10^−4^ and 9.8 × 10^−3^) despite the fact that they have differing patterns of cellular localization - one isoform localizes to the cytoskeleton, the other to the cytoplasm. ACP1 interacts with many proteins implicated in tumor progression, including janus kinase [26], 31 β-catenin [27], and the ephrin A2 receptor (EPHA2, also implicated in our core network, [28]), among others, and ACP1 was one of eight genes whose combined expression in peripheral blood has been suggested as a predictive signature of stage I lung adenocarcinoma [29]. The oncogenic relevance of the other four hub genes – including the heat shock protein HSP90AA1 [30–32], the lymphoid-enhancer binding factor LEF1 [21, 33–35], the DNA mismatch repair gene MLH1 [36–38], and the tumor suppressor RNA-binding motif protein RBM5 [39–41] - have been reviewed elsewhere [30–32, 36–41], further supporting this network as a molecular link between air pollution exposure and lung cancer risk.

Though the most prominent and consistent findings emerging from our analysis delineate network submodules implicated in cancer pathogenesis, the expression signatures that emerged also included a large collection of genes implicated in other diseases, including myocardial and cerebral ischemic injury, sudden cardiac death, and chronic obstructive pulmonary disease, particularly STAT1 [42–44], FZD2 [45, 46], GCLM [47], CD63 [48], and SP4 [49]. Given that all of these genes were members of the core gene expression set (Additional file 2: Table S1), and many were among the most highly connected hub genes, they represent important biological targets in the pathogenesis of these most common pollution-related diseases. Although the study was not designed to assess relationships with clinical disease, we applied a disease connectivity analysis using the “set analyzer” tool in the Comparative Toxicogenomic Database (http://ctdbase.org) and the list of 248 genes identified from our study (Additional file 3: Table S2). Of 20 diseases with Bonferoni-corrected *p*-values < 0.01, 12 of the were in the categories of cancer, lymphatic disease, and immune system diseases, consistent with our analysis using a gene-expression based network analysis.

Evaluation of strengths of our findings and their contribution to our current understanding of the adverse consequences of air pollution must consider several important strength and limitations relative to prior work. Unlike prior studies, measures of exposure were collected for all study subjects in real-life work settings over a workweek, providing more accurate estimates for analysis. Importantly, the exposure levels experienced by these workers (such as the truck drivers) overlap with ambient exposures experienced by the general public who would be likely to experience similar on-road exposures during such activities as commuting, making our results applicable to a wider population. Our repeated measures design provides for more accurate estimates of gene expression compared to single time-point studies, and is more robust to outliers. In addition, our sampling was performed at 10 sites within the Northeastern United States, providing good representation across the range of pollutant exposures. Lastly, we note the strong evidence of enrichment of our core gene sets in two previously published studies, providing strong evidence of the reproducibility and generalizability of our findings to other populations.

Several limitations, however, must be recognized. First, there are some limitations in the study design. Due to the demographics of employment of the trucking industry in the Northeast, our analysis was restricted to white men. The levels of physical activity during work, which differ between job titles, was not available. Heterogeneity in cell compositions, such as white blood cell counts, could not be addressed due to lack of data. We did examine the white blood cell marker genes based on the *cellmix* R package [50], and found no associations with exposures, either at gene level or gene set (pathway) level. Therefore, we do not believe that cell composition would bias our results. Second, the number of individual involved (63) was small, and the study might be underpowered, even with the repeated design. Third, although we examined the impacts of pollution in an occupational setting, the levels of pollution were low, likely due to decreasing pollution emissions in the trucking industry in recent years [51], and these low levels may explain the relatively modest number of observed changes in gene expression in our study. Therefore, we may not have detected additional genes of importance. Our reliance on GSEA mitigates this concern somewhat, but not completely. Lastly, our analysis relies on a one-week sampling of both exposures and expression measures, with the assumption that these observations are representative of more long-term processes. Though longer, more repeated sampling designs might provide more representative findings, two lines of evidence suggest that the efforts to collect such data would add only incrementally. First we have previously demonstrated in this industry that short windows (one week) of exposure sampling are representative of exposures measured at other time periods [52]. On average, our study subjects have been employed in the same position for 19 years. It is thus likely that the exposure estimates generated from this study are similar to what would be observed over longer periods of time (months to years). Additionally, we found no evidence of cross-week differential expression, providing some reassurance that the gene expression measures reflect the individual global patterns of gene expression measured over longer time periods. Finally, our finding that our core set of pollution-correlated genes was also prominent in two independent (albeit limited) datasets, suggests that our results may be generalizable and relevant to other exposed populations.

## Conclusions

In summary, we have characterized the molecular impact of traffic-related air pollution, and have identified a sub-network of interconnected genes implicated in cancer pathogenesis and related processes that are consistently perturbed in response to air pollution exposure. These data provide greater insights into the adverse health consequences of traffic-related air pollution.

## Additional files

Additional file 1: QQ plots after permutation for gene-level analysis. (PDF 4315 kb)
Additional file 2:
**Table S1.** List of the core set of 262 genes from differential expression analysis and GSEA. (DOCX 15 kb)
Additional file 3:
**Table S2.** List of enriched diseases in the Comparative Toxicogenomic Database using the core set of 248 genes from differential expression analysis and GSEA. (DOCX 14 kb)

## Abbreviations

BMI: Body mass index
COPD: Chronic obstructive pulmonary disease
DNA: Deoxyribonucleic acid
EC: Elemental carbon
FDR: False discovery rate
GEO: Gene Expression Omnibus
GSEA: Gene set enrichment analysis
OC: Organic carbon
P&D: Pick-up and delivery
PM_2.5_: Particulate matter ≤ 2.5 microns in diameter
QC: Quality control
RNA: Ribonucleic acid
SD: Standard deviation
