# Supplementary figures and images for: Gene expression network analyses in response to air pollution exposures in the trucking industry
Source: Environ Health. 2016 Nov 3;15:101. doi: 10.1186/s12940-016-0187-z (PMC5093980; doi:10.1186/s12940-016-0187-z)

**PM2 Permutation, lambda=1.152**

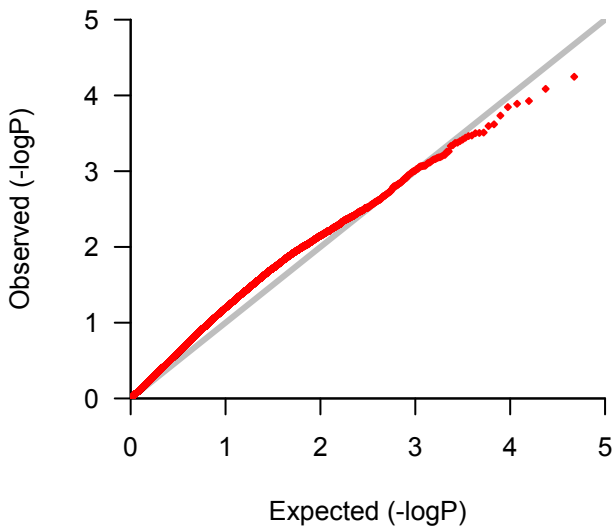

**OC Permutation, lambda=1.395**

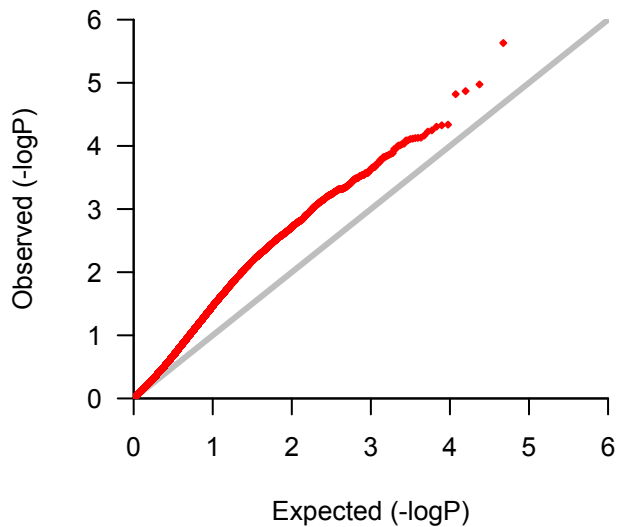

**EC Permutation, lambda=1.092**

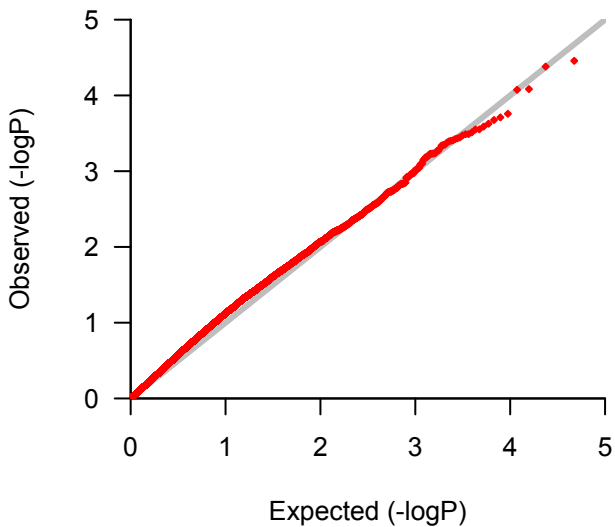

Supplement: Additional file 1: — QQ plots after permutation for gene-level analysis. (PDF 4315 kb) [file 12940_2016_187_MOESM1_ESM.pdf]
