# Supplementary material for: Gene expression network analyses in response to air pollution exposures in the trucking industry
Source: Environ Health. 2016 Nov 3;15:101. doi: 10.1186/s12940-016-0187-z (PMC5093980; doi:10.1186/s12940-016-0187-z)
Supplement: Additional file 2: — Table S1. List of the core set of 262 genes from differential expression analysis and GSEA. (DOCX 15 kb) [file 12940_2016_187_MOESM2_ESM.docx]

Additional file 2: Table S1: List of the core set of 262 genes from differential expression analysis and GSEA

| Category | Genes |
| --- | --- |
| Genes that were enriched in at least 20 sets common to all three pollution types (67) | ITK LEF1 PRIM1 DNAJC9 PDE4D CD28 CTPS GMPS TXK SLAMF1 PHF10 NOLC1 METTL3 LPIN1 GADD45A SLC20A1 **MOAP1** CCT4 COPS6 CYC1 CSTF3 RPIA PRKCH SNRPA1 MBNL2 RIB1 BLM NDUFV1 PUF60 DDX3X POLR3E INPP4B STAMBPL1 WEE1 UFSP2 SIDT1 OXCT1 FNBP4 TMEM97 DRG2 DNAJC11 COX11 USP34 AP3M2 UQCRC1 NGRN IPO5 YBX1 HSPA8 PDXK HSPA9 NDUFS3 TFDP2 PPP3CC ZBTB25 RRP1B CSNK2B FAM117A FOXO1 PPIA SQLE PHC1 NUP133 FHL1  CLPX RUVBL2 PSMB4 |
| Genes that were enriched in at least 10 gene sets in C2 (2) | CITED2 PHGDH |
| Genes that were enriched in at least 10 gene sets in C3 (5) | ZEB2 ETS1 **SP4** DDX3Y NLK |
| Genes that were enriched in at least 10 gene sets in C4 (8) | PSMB6 PSMD8 PPP2R1A WDR19 METAP1 EML3 FPR2 XPO6 |
| Genes that were enriched in at least 10 gene sets in C7 (63) | PIP4K2A GIT2 ICOS DENND2D RMND5A XBP1 TRIM32 ESYT1 MLH1 EDEM1 GNL2 DOCK9 MCTP2 CAPRIN2 MPHOSPH9 GNAQ DGAT1 RCAN3 APPL2 FAM172A TXN2 RCL1 RPL22 STK38 ZFP161 NHP2 GOT1 MDC1 LRMP MLF1 SIVA1 TECR RBM5 SASH3 KIAA1467 DENND5A ABHD14A MYB EPHA4 GLS KBTBD2 FGFR1OP ZCCHC11 ADH5 PNMA1 HADH TARBP1 AKAP8 EPHX2 CUEDC2 PAPOLG FBXL12 REXO2 CHD7 PGP SEL1L3 TMEM39A PIK3R5 ASTE1 YPEL2 AGPAT5 ACPP QSOX2 |
| Top 20 differentially expressed (DE) genes in OC (Total number of DE genes=260) | NT5DC2 SIL1 NUDT18 C9ORF7 PHF19 ZNF593 MFSD7 IFITM1 ODF3B LILRB4 MGC29506 ZDHHC24 SERPING1 IGLL1 MANBAL P2RX7 BST2 STAT1 C6ORF125 TMEM219 |
| Top 20 differentially expressed genes in EC  (Total number of DE genes=48) | LDHC **CLK1** NUDT7 CTAGE5 HSP90AA1 C9ORF30 UGP2 **CCNDBP1** C2ORF25 ATP5F1 **C9ORF106** BIRC2 TIPRL C1ORF103 **CNIH** GCOM1 PPP2R5C CDC14B MAPKSP1 ARMCX6 |
| Top 20 differentially expressed genes in PM2.5 (Total number of DE genes=49) | LYZ ZNF589 **APLP2** POL3S MEOX1 UCA1 PMS2L3 PSORS1C3 IGF1R FLJ38969 **NBPF1** NDRG3 C1QTNF6 RUNX1 LYZ CACNA1H SERP2 IFT52 USP36 HEBP1 |
| Genes that were differentially expressed by two or more pollution types (67) | **APLP2** HEMGN ERAP2 ACP1 **NBPF1** **CLK1** **CCNDBP1** **C9ORF106** **CNIH** OGDH CD2AP MTO1 CCT6A DHX15 RBM16 STRAP GLT25D1 ADSS MOCS2 CTR9 CYTIP IKZF5 FZD2 HLA-DMA GCLM AASDHPPT FAM10A7 TAOK2 G3BP2 GPAM UBE2H GFM2 **SP4** ZMYM5 TSGA10 MAN1A1 LSM12 C17ORF59 CD63 ZC3H14 ZFY STUB1 SIGMAR1 JMJD1C CDKN2AIP CLN3 VPS54 KLF3 MRPL45 TRIM23 LOC730268 SEC61A1 IVNS1ABP ELA1 SUV420H1 DYRK1A **MOAP1** HS.356079 C10ORF61 MPST LOC100133999 FOLR2 GPBAR1 TCP1 CNOT7 WRB TSGA14 |

Note: 1. No gene was enriched in at least 10 gene sets in category C5 or C6

2. The genes highlighted in bold text were included in two or more categories
