# Supplementary material for: Gene expression network analyses in response to air pollution exposures in the trucking industry
Source: Environ Health. 2016 Nov 3;15:101. doi: 10.1186/s12940-016-0187-z (PMC5093980; doi:10.1186/s12940-016-0187-z)
Supplement: Additional file 3: — Table S2. List of enriched diseases in the Comparative Toxicogenomic Database using the core set of 248 genes from differential expression analysis and GSEA. (DOCX 14 kb) [file 12940_2016_187_MOESM3_ESM.docx]

Additional file 3: Table S2: List of enriched diseases in the Comparative Toxicogenomic Database using the core set of 248 genes* from differential expression analysis and GSEA

| Disease Name | Disease ID | Disease Categories | P-value | Corrected P-value |
| --- | --- | --- | --- | --- |
| Neoplasms | MESH:D009369 | Cancer | 1.47E-12 | 7.29E-10 |
| Immune System Diseases | MESH:D007154 | Immune system disease | 1.50E-10 | 7.42E-08 |
| RNA Virus Infections | MESH:D012327 | Viral disease | 3.21E-09 | 1.59E-06 |
| Immunologic Deficiency Syndromes | MESH:D007153 | Immune system disease | 8.15E-09 | 4.04E-06 |
| Digestive System Diseases | MESH:D004066 | Digestive system disease | 8.24E-09 | 4.09E-06 |
| Sexually Transmitted Diseases | MESH:D012749 | Bacterial infection or mycosis\|Urogenital disease (female)\|Urogenital disease (male)\|Viral disease | 1.89E-08 | 9.38E-06 |
| Sexually Transmitted Diseases, Viral | MESH:D015229 | Viral disease | 1.89E-08 | 9.38E-06 |
| HIV Infections | MESH:D015658 | Immune system disease\|Viral disease | 2.23E-08 | 1.10E-05 |
| Lentivirus Infections | MESH:D016180 | Viral disease | 2.23E-08 | 1.10E-05 |
| Retroviridae Infections | MESH:D012192 | Viral disease | 2.23E-08 | 1.10E-05 |
| Virus Diseases | MESH:D014777 | Viral disease | 2.42E-08 | 1.20E-05 |
| Lymphoproliferative Disorders | MESH:D008232 | Immune system disease\|Lymphatic disease | 6.79E-08 | 3.37E-05 |
| Lymphatic Diseases | MESH:D008206 | Lymphatic disease | 1.70E-07 | 8.45E-05 |
| Neoplasms by Site | MESH:D009371 | Cancer | 1.81E-07 | 8.96E-05 |
| Neoplasms by Histologic Type | MESH:D009370 | Cancer | 2.48E-07 | 1.23E-04 |
| Immunoproliferative Disorders | MESH:D007160 | Immune system disease | 3.15E-07 | 1.56E-04 |
| Leukemia | MESH:D007938 | Cancer | 1.05E-06 | 5.19E-04 |
| Leukemia, Lymphoid | MESH:D007945 | Cancer\|Immune system disease\|Lymphatic disease | 2.54E-06 | 0.00126 |
| Gastrointestinal Diseases | MESH:D005767 | Digestive system disease | 5.55E-06 | 0.00275 |
| Digestive System Neoplasms | MESH:D004067 | Cancer\|Digestive system disease | 5.92E-06 | 0.00294 |

*14 out of 262 genes from Supplementary Table 1 could not been found in the CTD set analyzer.
